# Towards practical biocatalytic Baeyer-Villiger reactions: applying a thermostable enzyme in the gram-scale synthesis of optically-active lactones in a two-liquid-phase system

**DOI:** 10.1186/1860-5397-1-10

**Published:** 2005-10-07

**Authors:** Frank Schulz, François Leca, Frank Hollmann, Manfred T Reetz

**Affiliations:** 1 Max-Planck-Institut für Kohlenforschung, Kaiser-Wilhelm-Platz 1, D-45470 Mülheim/Ruhr, Germany

**Keywords:** Baeyer-Villiger oxidation, monooxygenases, two-liquid-phase system, stereoselective catalysis, biocatalysis

## Abstract

Baeyer-Villiger monooxygenases (BVMOs) are extremely promising catalysts useful for enantioselective oxidation reactions of ketones, but organic chemists have not used them widely due to several reasons. These include instability of the enzymes in the case of *in vitro* and even *in vivo* systems, reactant/product inhibition, problems with upscaling and the necessity of using specialized equipment. The present study shows that the thermally stable phenylacetone monooxygenase (PAMO) and recently engineered mutants can be used as a practical catalysts for enantioselective Baeyer-Villiger oxidations of several ketones on a preparative scale under *in vitro* conditions. For this purpose several parameters such as buffer composition, the nature of the solvent system and the co-factor regeneration system were optimized. Overall a fairly versatile and efficient catalytic system for enantioselective laboratory scale BV-oxidations of ketones was developed, which can easily be applied even by those organic chemists who are not well versed in the use of enzymes.

## Introduction

First reported in 1899, the Baeyer-Villiger (BV) reaction of ketones with formation of esters or lactones has become a fundamental and useful reaction in organic synthesis.[1–11] The practical value of these products for a variety of applications in the fields agrochemicals and pharmaceuticals has ever since driven the development of catalysts and reagents for this type of transformation. Catalytic routes have been reported using transition metals, [3,6,12] flavins,[13–15] and biocatalysts – the so-called Baeyer-Villiger monooxygenases (BVMOs).[7–11,16–26] Especially BVMOs are particularly interesting as they often combine high stereoselectivity with environmentally benign reaction conditions. The first BVMO to be identified was cyclohexanone monooxygenase (CHMO).[10] Following the pioneer work of Taschner regarding the application of CHMO as a stereoselective biocatalyst in organic synthesis,[23] this BVMO is still the most commonly used enzyme of its type.[16–18,22,24–30]

Despite the promising characteristics and almost 30 years of research on their biochemistry, BVMOs have not found widespread acceptance as enantioselective catalysts for laboratory-scale organic synthesis.[31–32] First, BVMOs are confined to aqueous reaction media within which most synthetically interesting substrates are poorly soluble. As a result, in most cases only low space-time yields are achievable. Furthermore, BVMOs are cofactor-dependent enzymes, i.e., they require stoichiometric amounts of costly and unstable nicotinamides (NAD(P)H) for reductive O_2_-activation.[33–35] Another challenge is the cost-factor of the BVMOs themselves, since their use usually requires tedious purification steps. These complications are frequently addressed by performing biocatalytic BV-oxidations *in vivo*, i.e., using whole, metabolically active microbial cells.[27–29] Whole-cell biocatalysis, however, has some serious drawbacks such as the necessity to use specialized personnel and equipment which may not be a problem in industry, but certainly is for chemists in most academic laboratories. Moreover, yields are often low due to substrate- and product toxicity and undesired reactant metabolism.[30] Thus, organic chemists are often reluctant to use BVMOs as useful catalysts when planning synthetic routes.

We therefore conclude that in the case of BVMOs, at least on mid-term basis, only *in vitro* biocatalysis has the potential of achieving true preparative relevance for the majority of organic chemists. This includes those who want to apply BVMOs only occasionally. En route to the goal of rendering BVMOs truly practical catalysts, various challenges have to be met. Often, BVMOs are specific for their natural substrate resulting in poor or even no activity with other compounds. This problem, however, can be considered to be solved as now various genetic tools are at hand with which enantiodiscrimination and the substrate spectrum of an enzyme can be controlled.[36–38] Along these lines we recently reported the directed evolution of stereoselectivity of CHMO for substrates which are oxidized with poor enantioselectivity when using the wild-type (WT) enzyme.[39–40] A great challenge emerges with the necessity to increase the efficiency of the BVMOs, specifically in terms of space-time yields and cost efficiency of the enzyme and cofactor. In particular the solubility of the hydrophobic substrate needs to be increased while preserving activity and stability of the biocatalyst under the unnatural reaction conditions. Moreover, BVMOs as isolated enzymes are very unstable and require special care in production and handling.[41–42] Finally, organic solvents have an adverse effect on stability, yet they may well be necessary in order to reach high space-time yields.

In the present contribution we address the aforementioned limitations and report the preparative-scale enantioselective BV-oxidation of *rac*-bicyclo [3.2.0]hept-2-en-6-one (**1**) and 2-phenylcyclohexanone (**5**) in a way that any synthetic organic chemist can perform. In particular we demonstrate that a BVMO can be stabilized in an aequeous-organic two-liquid phase medium under reaction conditions with high concentrations of several substrates.

We chose phenylacetone monooxygenase (PAMO) as the BVMO, which was first reported by Fraaije, Mattevi and co-workers in 2004.[43–44] Its thermostability renders PAMO a promising candidate in the development of robust and economically attractive BVMO-based reactions, even though it has a narrow substrate range. This problem was solved to some extent by rational redesign of the WT-PAMO with formation of P1, P2 and P3 mutants showing an altered substrate profile.[45] Thus, PAMO was turned into a "Phenylcyclohexanone Monooxygenase" (PCHMO), accepting not only 2-phenylcyclohexanone but also some other substrates.[45] In the present study we demonstrate the practical application of WT- and the engineered PAMOs for organic synthesis. Particularly, we evaluate the scope and limitations of both *in vivo* and *in vitro* biocatalysis using the PAMO mutants (PCHMOs) currently available in our laboratory. A general strategy for the practical biocatalytic preparation of enantiopure lactones based on BVMOs is proposed.

## Results and Discussion

The commercially available *rac*-bicyclo [3.2.0]hept-2-en-6-one (**1**) is a common benchmark substrate for BVMOs. It is readily oxidized by CHMO in an enantioselective way and the products of this oxidation are valuable intermediates in prostaglandin synthesis.[31–32,46]

**Scheme 1:**
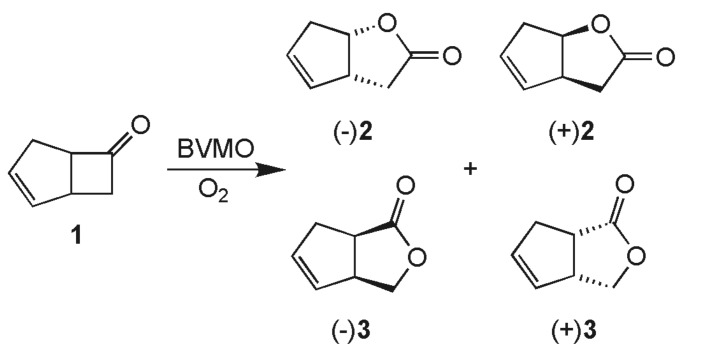


We first evaluated the stereoselectivity of the oxidation of **1** in standard whole-cell catalysis using WT-PAMO and the three mutant enzymes (P1-P3) recently reported by our group (Scheme 1).[45]

For the WT-PAMO we found after a reaction time of 24 h a conversion of 43% and moderate enantioselectivity for lactones (-)**2** and (+)**3** (Table 1). Conversion was drastically improved with mutants P1, P2, and P3 to more than 95% under the same whole-cell conditions, which clearly indicates enhanced enzyme activity. Additionally for P2 and P3 high enantiopurities for both product lactones were achieved. This result is reminiscent of the previous observation that these PAMO-mutants show an enhanced efficiency and stereoselectivity in oxidizing certain cyclic ketones, relative to WT-PAMO.[45]

**Table 1:** Activity of PAMO and PAMO-mutants in the BV-oxidation of bicyclo[3.2.0]hept-2-en-6-one (**1**) at a substrate concentration of 1 g/L in whole-cell catalysis.

Enzyme	Conversion after 24 h (%)	Product *ee* [%]	

		(-)**2**	**3**
		
WT-PAMO	43	92	48 (+)
P1-PAMO	>95	80	99 (-)
P2-PAMO	>95	93	>99 (-)
P3-PAMO	>95	92	>99 (-)

**Figure 1:**
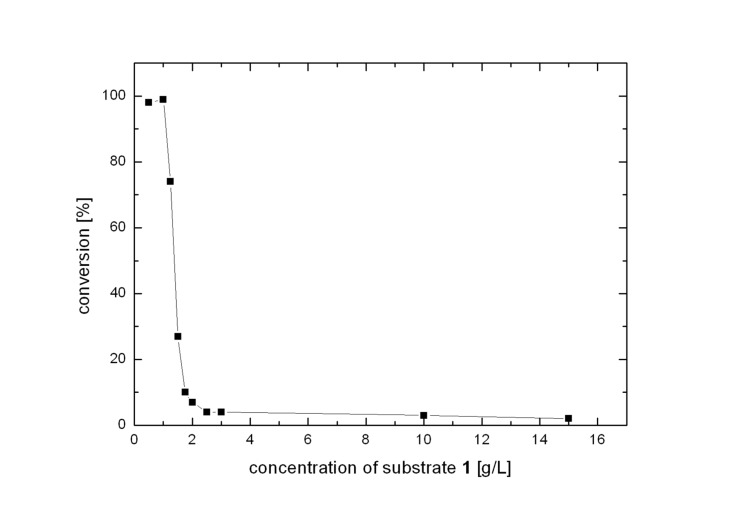
Evolution of conversion of ketone **1** using PAMO mutant P3 with increasing substrate concentration in whole-cell catalysis.

Initially these experiments were performed with a low concentration of substrate **1**. Unfortunately, a drastic decrease in conversion was observed when the substrate-concentration was increased to more than 1 g/L when using PAMO mutant P3 (Figure 1). A plausible explanation is substrate and/or product inhibition as reported earlier in the case of CHMO-catalyzed oxidation of the same substrate.[31]

In order to overcome this inhibition we tested the use of a second liquid phase in whole-cell catalysis using a water-immiscible organic phase to serve as substrate reservoir and product sink. Full conversion could be obtained using dioctylphthalate as the organic phase (medium: dioctylphthalate = 1:1) with substrate levels up to 3 g/L of **1**. However, further increases led to diminished conversion, which again is a sign of substrate and/or product inhibition. Significant improvement can only be expected using more polar organic phases which allow more favorable partitioning of the reactants between the aqueous and organic phase. Such organic solvents, however, are not compatible with whole-cell catalysis.[47]

We therefore evaluated an *in vitro* catalysis approach, initially using phenylacetone (**4**) as the substrate. Such catalysis using monooxygenases necessitates efficient recycling of the redox-cofactor NADPH. For this purpose a number of systems have been described. Indeed, several enzymes for recycling systems are commercially available.[48–50]

**Scheme 2:**
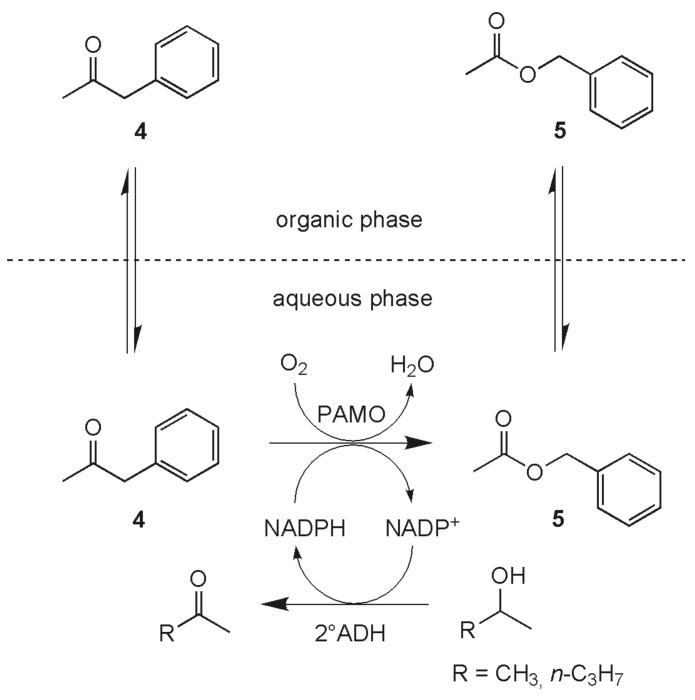


For efficient BV-oxidation catalysis with PAMO, the regeneration system must fulfill several requirements. The enzyme needs to be easily available. For this purpose an *E. coli* expression system would be appropriate due to the ease of handling this microorganism. The recycling enzyme must be thermostable at least to the extent of the production enzyme PAMO, and in addition it must exhibit a high degree of tolerance towards organic solvents. We speculated that thermostable alcohol dehydrogenases may meet these demands and decided to use the secondary alcohol dehydrogenase (2°ADH) from *Thermoanaerobacter ethanolicus*.[51–56] This 2°ADH oxidizes secondary alcohols like isopropanol to the corresponding ketones, thereby recycling one equivalent of NADPH which can then be utilized by PAMO in a coupled reaction (Scheme 2). Purification of this enzyme from an over-expressing *E. coli* strain proved to be straightforward, simple heat-treatment being sufficient to purify it up to near homogeneity.[56]

**Figure 2:**
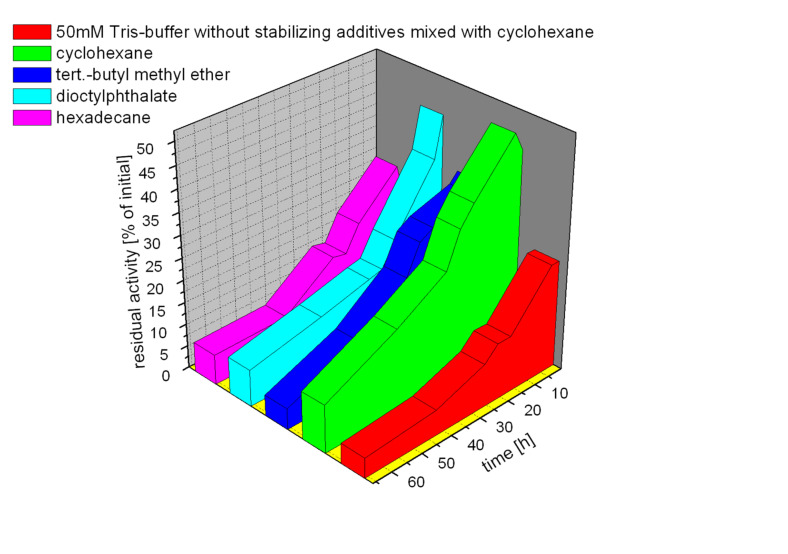
Effect of different solvents on the stability of PAMO, measured as residual activity. For cyclohexane as second phase values in 50 mM Tris-HCl and in optimized standard buffer are given, the results for the other solvents are shown only using the standard buffer. The first data points represent the residual activity after five minutes.

In order to stabilize the production enzyme in the presence of an organic phase, we evaluated various buffer additives such as sugars, non-ionic detergents, and bovine serum albumin (BSA) which are known to exert beneficial effects on other enzymes.[57] The most pronounced effect on the stability of the enzyme resulted from the use of detergents. Overall we found a buffer consisting of 50 mM Tris-HCl (pH 8.0 at 40°C), 2 g/L BSA, 5% (w/v) glucose, 5% (w/v) lactose, and 0.1% (v/v) Tween-20 to be optimal in maintaining PAMO stability. The influence of different solvents was then assayed in a 1:1 mixture under the reaction conditions envisaged (40°C, vigorous mixing). From the given selection of solvents, cyclohexane proved to be most suitable, exhibiting moderate influence on PAMO-stability, and methyl *tert*-butyl ether (MTBE) was found to be second best. However, in the case of cyclohexane, 50% of the initial enzyme activity is lost during the first five minutes of incubation (Figure 2). For the other solvents this effect is even more pronounced. After this initial short period the loss of activity of the enzyme proceeds much slower. The 2°ADH showed less dependency of its activity on the solvent and buffer composition and is generally more stable than PAMO. Interestingly, no apparent correlation can be drawn between the hydrophobicity of the solvent (expressed as logP) and the PAMO activity and stability, as was found in the case of whole-cell catalysis.[47] This is in agreement with results found previously for other systems.[58]

Optimization of the reaction conditions was then performed using the cheap phenylacetone (**4**) and WT-PAMO as a model system. Our goal was to develop a simple procedure allowing any organic chemist to handle an enzymatic catalysis with the BVMO using standard laboratory equipment and without special training in, for example, sterile working techniques. We concluded that it may be possible to use normal glassware, a magnetic stirring bar and an oil-bath to run the reactions. In addition, the enzymes should be easy to handle without cooling or other special precautions. To do so, we used the clarified lysate of the *E. coli*-cells and heat-treated it at 50°C for 1 h. Following centrifugation to again clarify the lysate, the resulting enzyme solution was used without further purification. The fraction of PAMO of the total protein was estimated after gel-electrophoresis by Coomassie-staining to be around 35%, the total protein concentration as measured by a Bradford assay was 800 μg/mL, resulting in a specific activity of 3.75 U/mg of total protein (1 U corresponds to the amount of enzyme that consumes 1 μmol/min of NADPH at 25°C). For the 2°ADH we proceeded in a similar way and purified the enzyme by heat-treating the *E.coli*-cells resuspended in Tris-buffer at 85°C for 15 min, followed by 15 min at 72°C, and afterwards centrifuged as above. The resulting enzyme solutions can be stored at room temperature for days or at 4°C for weeks without significant loss of activity. For long-term storage aliquots of the enzyme solution were frozen at -80°C.

The setup for the initial catalysis experiments was a glass-flask in each case, well ventilated with air and equipped with a magnetic stirrer and a reflux-cooler to prevent evaporation of the solvent. A reaction temperature of 40°C was chosen as a compromise between enzyme and NADPH-cofactor stability on the one hand, and high enzyme activity on the other. In the reaction we used significantly higher NADPH-regeneration activity (4 U/mL of 2°ADH) than PAMO-activity (0.6 U/mL) to force the equilibrium of NADPH/NADP^+^ to be on the reduced side. Thus, not just kinetic inhibition of the desired oxygenation reaction due to NADPH-limitation was circumvented. The stability of the cofactor itself was also increased, since NADP^+^ is rather unstable under basic conditions.[59]

Isopropanol, being the most effective stoichiometric sacrificial electron donor, resulted in significantly decreased stability of both enzymes used. The maximum concentration of isopropanol under which both enzymes show optimal activity was found to be 5% (v/v). In order to enhance conversion, we added surplus reducing equivalents in the form of 2-pentanol as a sacrificial substrate. Thus, due to the more favorable partitioning coefficient of 2-pentanol between the aqueous and the organic phase, lower actual alcohol concentrations in the aqueous phase can be achieved. Overall, the system benefits from the two liquid-phase approach in two respects, first by circumventing inhibitory effects of the substrates/products on the enzyme production and second by avoiding negative effects of the alcohol. Following this protocol it was possible to perform the BV-oxidation of phenylacetone (**4**) with formation of ester **5** in concentrations up to 1 g/L with 80% conversion within 24 h.

**Scheme 3:**
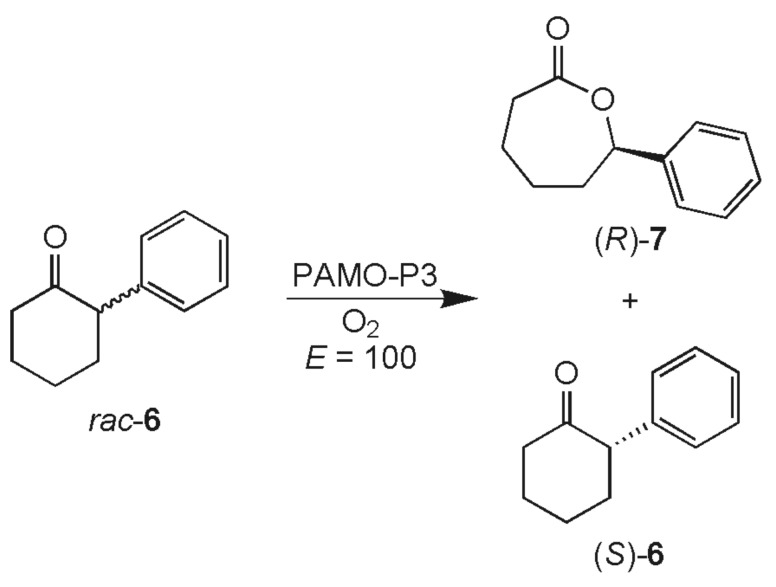


With this optimized protocol we evaluated the oxidation of ketones *rac*-bicyclo [3.2.0]hept-2-en-6-one (**1**) (Scheme 1) and *rac*-2-phenylcyclohexanone (**6**) (Scheme 3). As delineated above, both ketones are converted enantioselectively by mutant P3 in whole-cell catalysis (Table 1).

We were pleased to observe essentially identical enantioselectivities when applying *in vitro* catalysis (for ketone **1** compare Table 1; the kinetic resolution of ketone **6** is characterized by a selectivity factor of *E* = 100, the enantiopurity of lactone **7** being 95.4% *ee* (*R*)).

Ketone **1** could be quantitatively oxidized in a concentration of 5 g/L to the corresponding product lactones within 24 h, but at a concentration of 20 g/L conversion decreased to 50–60%. In this case the corresponding alcohol, bicyclo [3.2.0]hept-2-en-6-ol, was also obtained in up to 30% yield, presumably as the product of the reduction by 2°ADH. This opens up the opportunity to start the reaction not from the ketone but already from the corresponding alcohol in a more elegant way, thus circumventing the sacrificial alcohol.[60] Overall, we conclude that the originally observed reactant/product inhibition in whole-cell catalysis is no longer the problem.

The smooth BV-oxidation of ketone **6** at concentrations of 5 g/L caused no problems following a slight modification. In this case the reaction was found to be more effective when MTBE was used as the second phase, most likely due to the low solubility of the substrate in cyclohexane. The kinetic resolution reached the optimal 50% conversion after about 24 h. Upscaling to gram-quantities was straight-forward without changes in the procedure.

Overall we obtained turnover numbers (TONs) of more than 30000 for the P3-PAMO-catalyzed BV-oxidation of ketone **1**, which to the best of our knowledge is unprecedented for flavin-dependent monooxygenases in the presence of organic solvents (Table 2).

**Table 2:** Catalytic efficiency of WT- and P3-PAMO in *in vitro* catalysis with two-liquid phases. TF: Turnover frequency; TN: Total turnover number.

Substrate	Enzyme	Substrate concentration	TF (h^-1^) (BVMO)	TN (BVMO)	TN (NADP^+^)	Reaction scale

**1**	P3-PAMO	5 g/L	313	37640	400	20 mL
**4**	WT-PAMO	1 g/L	98	4715	12^a^	20 mL
**6**	P3-PAMO	5 g/L	394	9471	23.3^a^	200 mL

^a^ The total turnover number for NADP^+^ can be increased up to 400 also for substrates **4** and **6**. This was tested only on an analytical 3 mL scale and is therefore not included in this table.

**Figure 3:**
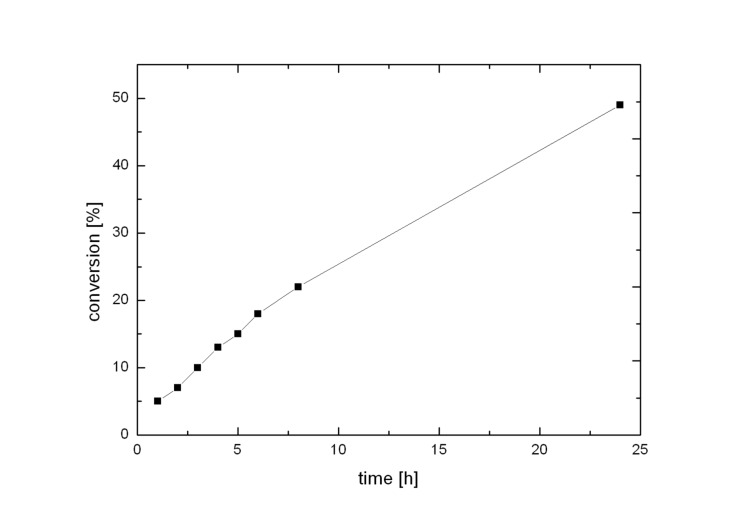
Conversion during the oxidation of ketone **6** in a two-liquid phase system over the time. The catalyst shows no decrease in activity for at least ten hours.

P3-PAMO was found to be active for more than ten hours in the reaction without any loss of activity (Figure 3) and to exhibit reaction rates that correspond to those that were found previously in steady-state-kinetic analysis in the absence of organic solvents for ketone **6** as substrate.[45]

Therefore, the addition of the organic solvent does not significantly reduce the stability of the enzyme when the optimized buffer is used. This was not achieved previously with related systems and is especially not possible when using immobilized enzymes, a standard method for stabilizing them. We view this study as a crucial step towards practical BVMOs, because PAMO, the P1-P3 mutants and presumably future PAMO-variants can now be used in a simple preparative scale procedure.

We compared the system developed in this study to an analogous synthetic organocatalyst for the enantioselective BV-oxidation as described by Murahashi et al. in 2002.[13] The Murahashi system is related to PAMO because it also uses a flavin-derived catalyst within a chiral environment. Of course, the molecular weights of the catalysts differ vastly. However, the comparison shows that the PAMO-catalyzed BV-oxidation is in fact practical on the laboratory scale in terms of stereoselectivity, catalyst productivity and stability and finally also in the ease of carrying out such reactions (Table 3).

**Table 3:** Comparison of P3-PAMO with Murahashi's chiral bisflavin organocatalyst as a chemical model catalyst for enantioselective BV-oxidations.

	Murahashi's bis-flavine	P3-PAMO

Steps in catalyst synthesis	5	2^a^
Time required for synthesis of the catalyst	2.2 d^b^	<1.5 d
Yield of catalyst synthesis	55.4%	~0.2%^c^
g (catalyst)/g (product)	~0.5 g/1 g (based on 4-phenyl-dihydrofuran-2-one as product)	~0.044 g/1 g (based on lactones **2** as products)^d^
Substrate scope and stereoselectivity	4-membered cyclic ketones described; highest *ee*-value: 74%	cyclic and non-cyclic ketones, substrate scope can be engineered; highest *ee*-value: 99%
Turnover number	9	>30000
Turnover frequency	0.06 h^-1^	313 h^-1^
Oxidants used	H_2_O_2_^e^	O_2_, NADPH
Reaction conditions	CF_3_CH_2_OH/MeOH/H_2_O	buffer/cyclohexane (or MTBE)
Solvents/temperature	-30°C	40°C

^a^ One step for the production of each enzyme, PAMO and 2°ADH are counted. ^b^ Time demand is calculated on the basis of the reaction times as given in the publication. ^c^ Yield is based on the ingredients used for preparation of the bacterial medium: Yeast extract, peptone, and glycerol, calculated as mass-percent. ^d^ Total amount of protein as well as NADP^+^ are taken into account. ^e^ Recently a variant with reductive regeneration of the flavin catalyst has been reported, though not yet in an enantioselective version.[15]

## Conclusion

In conclusion, this study represents the first protocol for performing stereoselective biocatalytic Baeyer-Villiger oxidations in-vitro in an organic chemistry laboratory. Following standard fermentation techniques production of the enzymes is straighforward. Tedious purification steps of the enzymes proved to be unnecessary.

Further experiments concerning an alternative NADPH-recycling system are currently underway in our laboratory, specifically with the purpose of eliminating the undesired reduction of the substrate ketones. Future work will also focus on the directed evolution of PAMO-mutants with the aim of broadening further the range of substrate acceptance while maintaining high thermal stability. Truly practical and versatile catalysts for selective BV-reactions may then emerge, making a commercialization of these enzymes possible on the basis of the work presented here.

## Supporting Information

File 1 Experimental section.
