# Supplementary material for: Towards practical biocatalytic Baeyer-Villiger reactions: applying a thermostable enzyme in the gram-scale synthesis of optically-active lactones in a two-liquid-phase system
Source: Beilstein J Org Chem. 2005 Oct 7;1:10. doi: 10.1186/1860-5397-1-10 (PMC1399458; doi:10.1186/1860-5397-1-10)
Supplement: File 1 — Experimental section. [file Beilstein_J_Org_Chem-01-10-s001.doc]

**Experimental section:**

*Materials*:

All chemicals were purchased from Aldrich, Fluka, Acros, or Merck and used without further purification. Media components were purchased from Applichem or Invitrogen. NADPH and NADP^+^ were purchased from Jülich Fine Chemicals. The sugars were obtained from Sigma, Fluka or Applichem, the detergents from Applichem and Aldrich. Racemic standards for GC-analysis were synthesized by *m*CPBA-oxidation of the corresponding ketones.

*Preparation of PAMO and PAMO-mutants for in vitro catalysis:*

From an overnight pre-culture of *E. coli* TOP10 [pPAMO] in LB-medium (supplied with 100 μg/mL carbenicilline) 200 mL TB-medium (0.1% arabinose, 100 μg/ml carbenicilline) were inoculated and grown at 37 °C.Error: Reference source not found,Error: Reference source not found,Error: Reference source not found The cells were harvested and the pellet was briefly frozen at -80 °C and thawed. Then the pellet was resuspended in 50 mM Tris-HCl (pH 7.4) and the cells disrupted by sonication. The resulting lysate was centrifuged (15500 x g, 4 °C) and subsequently incubated at 50 °C for 1 h. After short incubation on ice, the lysate was again centrifuged and subsequently stored in aliquots at either 4 °C or -80 °C. The overall time demand for this procedure is less than 1.5 days. The PAMO activity was measured based on conversion dependent NADPH-depletion at 25 °C under substrate saturation conditions as described previously.Error: Reference source not found,Error: Reference source not found,Error: Reference source not found The protein concentration was determined using the Biorad Bradford assay with BSA as standard. The samples were analyzed by 10% SDS-PAGE. Before use the sample was incubated at 50 °C for 20 min to reactivate PAMO.

*Preparation of 2°ADH for in-vitro catalysis:*

From an overnight preculture of Dh5α[pADHB1M1-kan] in LB-medium (supplied with 100 μg/mL carbenicilline and 50 μg/mL kanamycine), 200 mL TB-medium (supplied with 100 μg/mL carbenicilline and 50 μg/mL kanamycine) were inoculated and grown at 37 °C without induction. The cells were harvested and resuspended in 50 mM Tris-HCl (pH 8.0). The suspension was frozen in liquid nitrogen and thawed at 50 °C. The resulting suspension was incubated at 85 °C for 15 min, subsequently at 72 °C for 15 min, then incubated on ice for 30 min. The suspension was centrifuged (15500 x g, 4 °C) and the resulting solution stored in aliquots at either 4 °C or -80 °C. The enzymatic activity was measured spectrophotometrically by NADPH-generation in the oxidation of isopropanol at 25 °C essentially as described in the literature.Error: Reference source not found The samples were analyzed by 10% SDS-PAGE and the protein concentration determined using the Biorad Bradford assay with BSA as standard. Before use the samples were incubated at 50 °C for 20 min to reactivate 2°ADH.

**Table 4**: Substances assayed as stabilizing additive (semi-quantitative weighing).

| Additives | Effect^a^ | Remarks |
| --- | --- | --- |
| Monosaccharides: glucose / xylose | + / 0 | Beneficial effects of sugars have been described for other enzymes, see ref. Error: Reference source not found |
| Disaccharides: saccharose / lactose | 0 / + |  |
| Sugar alcohols: sorbitol / mannitol | 0 / 0 |  |
| Bovine Serum Albumin (BSA) | + | For beneficial effects compare ref. Error: Reference source not found |
| Non-ionic detergents: Triton X-100, Tween-20, Tween-80 | - / +++/ ++ |  |
| Flavin-Adenin-Dinucleotide (FAD) | ++ | Stabilizing effect of FAD is described in ref. Error: Reference source not found |
| Catalase | 0 | Catalase might stabilize enzyme towards H_2_O_2_ |

^a^ 0 = no significant effect on enzyme stability, + - +++ = increasingly beneficial effect on stability, - = negative effect on stability.

50 mM Tris-HCl (pH 8.5 at room temperature, estimated pH at 40 °C about 8.0[[1]](#endnote-2)) was used in all cases. The assays were performed with 500 μL buffer including the enzyme preparation as described above and supplied with the additives of choice mixed with 500 μL of the organic solvent in an Eppendorf tube that was kept at 1400 rpm and 40 °C. Aliquots of 25 μL of the aqueous phase were taken and analyzed by NADPH-depletion for their residual activity as described.Error: Reference source not found,Error: Reference source not found,Error: Reference source not found

*Composition of the Standard buffer in the in-vitro catalysis experiments:*

- 50 mM Tris-HCl, pH 8.5 at 25 °C (corresponds to ca. pH 8.0 at 40 °C)
- 2 g/L BSA
- 5% (w/v) glucose
- 5% (w/v) lactose
- 0.1% (v/v) Tween-20

*In vitro-catalysis in the BV- oxidation of phenylacetone (****3****):*

In a flask, the following ingredients were successively added: 5 mL of standard buffer, 100 µL of NADP^+^-stock solution (final concentration = 0.5 mM), WT-PAMO solution (final activity = 0.6 U/mL), 2°ADH solution (final activity = 4 U/mL), then the volume of the aqueous phase was adjusted to 10 mL with standard buffer. Subsequently 20 µL of phenylacetone (**3**), 100 µL of isopropanol, 50 µL of 2-pentanol and 10 mL of cyclohexane were added. The reaction was heated at 40 °C, phase separation was achieved by centrifugation. A sample of the reaction was analyzed by GC after 3 days and 80% conversion was reached. GC-method: HP-5 column (30 m x 0.32 mm x 0.25 μm); 60 °C (2 min) – 30 °C/min - 250 °C (5 min).

*In vitro-catalysis in the BV- oxidation of rac-bicyclo[3.2.0]hept-2-en-6-one (****1****):*

The ingredients for the aqueous phase were mixed as described above for substrate **3**, subsequently 100 µL of isopropanol, 100 µL of 2-pentanol, 10 mL of cyclohexane and 100 µL (0.1025 g, 0.95 mmol) of *rac*-bicyclo[3.2.0]hept-2-en-6-one (**1**) were added. The reaction was heated at 40 °C. 95% conversion of starting material was reached after 4 days, separation of the two liquid phases was achieved by centrifigation. The stereochemistry of the products was assigned based on the literature.Error: Reference source not found GC-method: BGB-178 column (15 m x 0.25 mm x 0.25 μm); isothermal 115 °C.

*In vitro-catalysis in the BV-oxidation of 2-phenylcyclohexanone (****5****):*

In a 1 L flask, we added successively: Standard buffer, NADP^+^-stock solution (final concentration = 0.5 mM), P3-PAMO- and 2°ADH solution were mixed as described above to a final volume of 100 mL. Subsequently 1 mL of isopropanol, 1 mL of 2-pentanol, 100 mL of methyl *tert*-butyl ether, and 1 g (5.7 mmol) of 2-phenylcyclohexanone (**5**) were added. The mixture was heated at 40 °C and the progress of the reaction was followed by GC. The reaction was stopped after 24 h to reach 50% conversion. The mixture was centrifuged to facilitate separation of the two liquid phases. The organic solvent was removed under reduced pressure and the crude product was purified via silica-gel chromatography. (Eluent: pentane/ethyl acetate: 9/1). 475 mg (2.7 mmol, 47%) of 2-phenylcyclohexanone **5** and 444 mg (2.3 mmol, 40%) of the corresponding lactone (*R*)-**6** were isolated with an *ee* of 95.4%. GC-method: see reference.Error: Reference source not found

*Biphasic whole-cell catalysis in the BV-oxidation of rac-bicyclo[3.2.0]hept-2-en-6-one (****1****):*

*E. coli* TOP10 [pPAMO-P3]-expression cultures in TB-medium (supplied with 100 mg/L carbenicilline) were grown to an OD_600_ of 2.5-3.0 and subsequently supplemented with 5 g/L of glycerol. P3-PAMO was then heat-activated at 45 °C for 15 min.Error: Reference source not found Subsequently 20 mL of the culture were mixed with 120 µL (1.1 mmol) of *rac*-bicyclo[3.2.0]hept-2-en-6-one (**1**) and 20 mL of dioctylphthalate. The reaction was well mixed for 24 h at 37 °C and continuously supplied with air. 5 mL of the solution were extracted with 5 mL of ethyl acetate and the conversion was determined by GC analysis as described above.

1. . Calculation of thermodynamically correct pH buffers: [http://www.bi.umist.ac.uk/users/mjfrbn/buffers/makebuf.asp]. [↑](#endnote-ref-2)
